# Magnolol Induces the Extrinsic/Intrinsic Apoptosis Pathways and Inhibits STAT3 Signaling-Mediated Invasion of Glioblastoma Cells

**DOI:** 10.3390/life11121399

**Published:** 2021-12-14

**Authors:** Po-Fu Yueh, Yuan-Hao Lee, Chun-Yu Fu, Chun-Bin Tung, Fei-Ting Hsu, Keng-Li Lan

**Affiliations:** 1 Institute of Traditional Medicine, School of Medicine, National Yang Ming Chiao Tung University, Taipei 112, Taiwan; u409801001@ym.edu.tw; 2 Department of Biological Science and Technology, China Medical University, Taichung 406, Taiwan; 3 Department of Radiation Physics, Division of Radiation Oncology, The University of Texas MD Anderson Cancer Center, Houston, TX 77030, USA; chrisdanto@gmail.com; 4 Department of Colon and Rectal Surgery, Show Chwan Memorial Hospital, Changhua 500, Taiwan; 5 Division of General Surgery, Department of Surgery, Tri-Service General Hospital, National Defense Medical Center, Taipei 114, Taiwan; 6 Department of Emergency Medicine, Show Chwan Memorial Hospital, Changhua 500, Taiwan; 7 Department of Oncology, Taipei Veterans General Hospital, Taipei 112, Taiwan

**Keywords:** magnolol, apoptosis, STAT3, invasion, GBM

## Abstract

Glioblastoma multiforme (GBM) is the most common form of malignant brain tumor, with poor prognosis; the efficacy of current standard therapy for GBM remains unsatisfactory. Magnolol, an herbal medicine from *Magnolia officinalis*, exhibited anticancer properties against many types of cancers. However, whether magnolol suppresses GBM progression as well as its underlying mechanism awaits further investigation. In this study, we used the MTT (3-(4,5-Dimethylthiazol-2-yl)-2,5-Diphenyltetrazolium Bromide) assay, apoptosis marker analysis, transwell invasion and wound-healing assays to identify the effects of magnolol on GBM cells. We also validated the potential targets of magnolol on GBM with the GEPIA (Gene Expression Profiling Interactive Analysis) and Western blotting assay. Magnolol was found to trigger cytotoxicity and activate extrinsic/intrinsic apoptosis pathways in GBM cells. Both caspase-8 and caspase-9 were activated by magnolol. In addition, GEPIA data indicated the PKCδ (Protein kinase C delta)/STAT3 (Signal transducer and activator of transcription 3) signaling pathway as a potential target of GBM. Magnolol effectively suppressed the phosphorylation and nuclear translocation of STAT3 in GBM cells. Meanwhile, tumor invasion and migration ability and the associated genes, including MMP-9 (Matrix metalloproteinase-9) and uPA (Urokinase-type plasminogen activator), were all diminished by treatment with magnolol. Taken together, our results suggest that magnolol-induced anti-GBM effect may be associated with the inactivation of PKCδ/STAT3 signaling transduction.

## 1. Introduction

GBM, grade IV astrocytoma, is the most common tumor of the central nervous system (CNS) and is associated with poor prognosis among adults due to rapid growth and aggressive invasion [1,2]. Development of effective anti-GBM agents with less toxicity is crucial for improving survival of patients.

Herbal medicines have been recognized as potential complementary strategies for GBM [3]. Trogrlic et al. presented that a phytotherapy involving five types of herbal medicines was conducive to therapeutic and survival benefits of standard oncological treatment for GBM [4]. In addition, medicinal herbs were demonstrated to induce GBM regression in cell and animal models. It was found that bioactive compounds isolated from medicinal herbs mediate anticancer activities, such as cell cycle inhibition, antitumor immune responses, suppression of oncogenic signal pathways, autophagy and apoptosis [3,5,6,7].

Induction of cell apoptosis as well as inhibition of invasion-associated proteins by bioactive compounds mitigates growth and invasion of GBM [5]. For instance, capsaicin, quercetin and fucoxanthin have been shown to eliminate GBM cell growth by triggering the activation of death receptor (extrinsic)- and mitochondria (intrinsic)-dependent apoptosis [8,9,10]. The extrinsic and intrinsic apoptosis pathways are, respectively activated by death receptor-ligand interactions and intracellular stresses (i.e., DNA damage and endoplasmic reticulum stress). In response to extracellular and intracellular signals, respectively, caspase-8 and caspase-9 are initiator caspases that activate downstream executioner caspases to mediate cell death [11]. Sinomenine hydrochloride, a major bioactive compound derived from the medicinal herb *Sinomenium acutum*, has been indicated to reduce invasion ability of GBM cells by decreasing protein levels of matrix metalloproteinase-2/-9 (MMP-2/-9) [12]. STAT3, on the other hand, is an oncogenic transcription factor that promotes aggressive invasion of GBM by upregulating the expression of invasion-associated genes [13]. As increased activation of STAT3 contributes to the enhancement of invasion ability in GBM cells, STAT3 has been suggested as a potential target for suppression of GBM cell invasion [13,14].

*Magnolia officinalis* are Chinese herbal medicines widely used for the treatment of various disorders, such as gastroenterological disease, cough, headache, anxiety and allergy [15,16]. Magnolol, a bioactive compound extracted from *Magnolia officinalis*, can cross the blood–brain barrier to attenuate oxidative stress against ischemic brain damage [17]. Magnolol has been reported to inhibit cell proliferation and migration through inducing G1 cell cycle arrest and reducing N-cadherin expression in GBM cells [18,19]. However, the regulatory actions of magnolol on the apoptosis and STAT3-mediated invasion of GBM cells remains ambiguous. The main purpose of the present study was to verify whether initiation of extrinsic/intrinsic pathways and suppression of STAT3 signaling are associated with magnolol-induced apoptosis and invasion inhibition on GBM cells.

## 2. Materials and Methods

### 2.1. Chemicals, Antibodies, and Reagents

Magnolol was purchased from Wuhan ChemFaces Biochemical Co., Ltd. (Wuhan, Hubei, China). MTT and dimethyl sulfoxide (DMSO) were both obtained from Sigma Chemical Co. (St. Louis, MO, USA). Roswell Park Memorial Institute 1640 (RPMI 1640) medium, Dulbecco’s Modified Eagle Medium (DMEM), fetal bovine serum (FBS) and penicillin-streptomycin (PS) were purchased from GIBCO^®^/Invitrogen Life Technologies (Carlsbad, CA, USA). Primary antibodies against Death receptor 4 (DR4) (D9S1R) (#42533, Cell Signaling Technology, Danvers, MA, USA), Death receptor 5 (DR5) (D4E9) XP^®^ (#8074, Cell Signaling Technology), MMP-9 (PA5-13199, Invitrogen, Waltham, MA, USA), uPA (GTX79597, GeneTex, Irvine, CA, USA), PKCδ (Thr505) (#9374, Cell Signaling Technology), PKCδ (D10E2) (#9616, Cell Signaling Technology), STAT3 (Tyr705) (D3A7) XP^®^ (#9145, Cell Signaling Technology), STAT3 (124H6) (#9139, Cell Signaling Technology), Myeloid-cell leukemia-1 (MCL-1) (D35A5) (#5453, Cell Signaling Technology), and β-actin (sc-47778, Santa Cruz Biotechnology, Dallas, TX, USA) for Western blotting were all purchased from different companies as mentioned. Secondary antibodies, peroxidase affiniPure Goat Anti-Mouse IgG and Goat Anti-Rabbit IgG were purchased from Jackson Immunoresearch Laboratories Inc. (West Grove, PA, USA).

### 2.2. Cell Culture of GBM

GBM8401 cells were provided from professor Jing-Gung Chung at China Medical University, Taiwan. A stable GBMcell line, BP5 cells, was transformed from a GBM patient sample. This cell was received from professor Szu-Yi Chou in Taipei Medical University, Taiwan with IRB Protocol approval ID 201805040 [20]. GBM8401 cells and BP5 cells were maintained with RPMI 1640 and DMEM high-glucose medium (Gibco, Waltham, MA, USA) containing 10% FBS and 1% PS in 5% CO_2_ and 37 °C, respectively.

### 2.3. MTT Assay

The cell viability of GBM cells after magnolol treatment was measured by MTT assay. GBM8401 and BP5 cells were cultured in 96-well plates at 5000 cells per well for 18 h after that, and cells were treated with different concentration (0–200 μM) of magnolol for 24 and 48 h. To measure the viability, the MTT solution (final concentration = 0.5 mg/mL) was added into 96-well plate and incubated in 37 °C for 2 h. Then, we added 100 μL dimethyl sulfoxide (DMSO) to dissolve the formazan (purple crystal) and measured the absorbance wavelength with 570 nm; the blank value was defined as baseline (+/−0.01). The MTT assay was repeated for three times.

### 2.4. Flow Cytometry

GBM8401 cells were cultured in 6-well plates at 1 × 10^5^ cells per well for 18 h and treated with 0, 12.5, 25, and 50 μM of magnolol for 48 h. After treatment, cells were collected with trypsin and measured by FITC-DEVD-FMK (cleaved caspase-3), FITC-IETD-FMK (cleaved caspase-8), FITC-VAD-FMK (cleaved caspase-9), Fas-PE, Fas-FITC, DR4 (D9S1R), DR5 (D4E9) XP^®^, DIOC_6_ (for mitochondria membrane potential) and FITC Annexin V Apoptosis Detection Kit (BD Bioscience, Becton Drive Franklin Lakes, NJ, USA) for apoptosis detection, respectively. After cell-staining processes, the fluorescence signals from cells were identified by flow cytometry (NovoCyte, Agilent Technologies, Santa Clara, CA, USA). The activation of each apoptosis marker after magnolol treatment was quantified by FlowJo software (version 7.6.1; FlowJo LLC, Ashland, OR, USA). All flow cytometry experiments were repeated three times.

### 2.5. GEPIA Analysis

To identify the role of STAT3 and PKCδ on glioma patients (low-grade glioma and GBM), we used GEPIA (Gene Expression Profiling Integrated Analysis) software. The mRNA expression level of the STAT3 was measured by using the GEPIA platform. The overall survival analyses between high STAT3 and low STAT3 expression level were executed using the Kaplan–Meier curve in the GEPIA database. The Pearson Correlation Coefficient between STAT3 and PKCδ genes were also performed by GEPIA.

### 2.6. Transwell Invasion Assay

GBM8401 cells were cultured in 6-well plates at 1 × 10^5^ cells per well for 18 h, and treated with 0, 12.5, and 25 μM of magnolol for 48 h. After treatment, 1 × 10^5^ cells were collected with trypsin and resuspended with serum-free medium, placed into the upper chamber of 8 μm pore transwell (BD Biosciences) with matrigel (matrigel:medium = 1:1) pre-coated, and putted transwells in 24-well plates that contain 10% FBS medium, prior to incubation in 37 °C for 24 h. The transwells were fixed with fixation buffer (methanol:acetic = 3:1), and the cells were stained with 0.1% crystal violet for 15 min. Bright-field images of transwell membrane were obtained by bright field Nikon ECLIPSE Ti-U microscope (Tokyo, Japan). The number of invasion cells on transwell membrane were calculated by Image J software version 1.50 (National Institutes of Health, Bethesda, MD, USA). All transwell invasion assays were repeated two times.

### 2.7. Wound Healing Assay

Cells were cultured into a 6-well plate with 1 × 10^5^ cells per well for 18 h and treated with different concentrations of magnolol for 48 h. After treatment, 1 × 10^5^ cells were collected with trypsin and placed into two-well insert (Ibidi, Germany) overnight. Upon the removal of the two well inserts at the 0th and 21st hour time points, images of cell migration were obtained by bright field Nikon ECLIPSE Ti-U microscope and analyzed by Image J software version 1.50. All wound healing assays were repeated for two times.

### 2.8. Western Blotting Assay

For protein extraction, GBM8401 cells were cultured in the medium and treated with 0, 12.5, and 25 μM of magnolol for 48 h prior to cell lysis with RIPA buffer containing phosphatase and proteinase inhibitor cocktail. Cell lysate was separated by different percentage of SDS-PAGE gel (8–12%), while the separated proteins were further transferred onto polyvinylidene difluoride (PVDF) membranes from SDS-PAGE gel. Then, PVDF membranes were blocked with 5% non-fat milk at room temperature for one hour, and incubated the primary antibodies at 4 °C overnight and secondary antibodies at room temperature for one hour. The antibody-conjugated membranes were reacted with Immobilon Western Chemiluminescent HRP Substrate (Pierce, Rockford, IL, USA). The luminescence signal from each membrane of specific protein was then detected by the UVP ChemiDoc-ItTM (Analytik Jena, Jena, Germany), and their sample specific band intensities were quantified by VisionWorks (Analytik Jena, Jena, Germany). Quantification data were all normalized by housekeeping gene β-actin expression and averaged by three repeated experiments. All Western blotting assays were repeated for three times.

### 2.9. Translocation Assay

Cells were seeded on 2 mm × 2 mm coverslips at 1 × 10^5^ cells per well for 18 h and treated with 0, 12.5, and 25 μM of magnolol for 48 h. Cells on coverslips were fixed by 4% paraformaldehyde for 15 min and permeabilized with 0.1% Triton-X 100 for 15 min. Then, cells were blocked with 3% bovine serum albumen and stained with 1:200 Stat3 primary antibody (#9139, Cell signaling technology) at 4 °C overnight. The 1:200 Alexa Fluor^®^ 488 AffiniPure Goat Anti-Mouse IgG (H+L) secondary antibody and 1 μg/mL DAPI were administered for cell incubation at room temperature for one hour and two minutes, respectively. The stained coverslips were mounted onto glass slide with mounting buffer and stored in 4 °C protected from light. Images were obtained using DAPI and FITC channels with fluorescence microscopy (Revolve, ECHO, San Diego, CA, USA). The translocation assay was repeated two times.

### 2.10. Statistical Analysis

Tests of one-way and two-way ANOVA were used in this study for comparing groups of different concentration treatments using Microsoft Excel 2016. Western blotting, flow cytometry and transwell experiments were analyzed with Tukey’s test; the wound-healing assay was assessed with multiple comparisons and the Sidak’s method after tests of one-way and two-way ANOVA. *p*-values smaller than 0.05 were defined as a significant difference. Each value in this study is displayed as mean ± standard error. Statistical differences between groups were mentioned and displayed in figure legends.

## 3. Results

### 3.1. Magnolol Effectively Induced Cytotoxicity and Activated the Extrinsic/Intrinsic Apoptosis Pathways of GBM Cells

**Figure 1:**
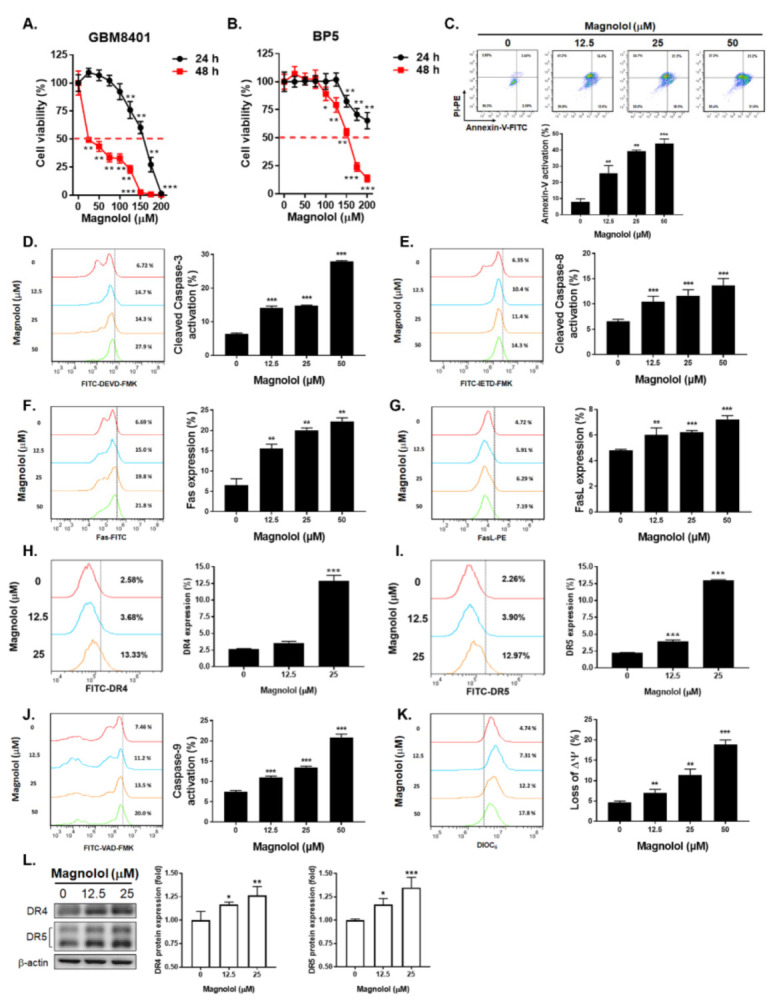
Cytotoxicity and apoptosis effects of magnolol on GBM cells. (**A**) GBM8401 cells and (**B**) BP-5 cells are treated with 0–200 μM magnolol for 24 and 48 h. GBM8401 cells are treated with 0, 12.5, 25, 50 μM magnolol for 48 h and assayed by (**C**) annexin-V/PI, (**D**) FITC-DEVD-FMK, (**E**) FITC-IETD-FMK, (**F**) Fas-FITC, (**G**) Fas-L-PE, (**H**) DR4, (**I**) DR5, (**J**) FITC-VAD-FMK and (**K**) DIOC_6_ staining, respectively. (**L**) DR4 and DR5 protein expressions are performed by Western blotting. Full Western blotting images have been provided in supplementary material (Material S1). (* *p* < 0.05, ** *p* < 0.01 and *** *p* < 0.005 vs. 0 μM magnolol).

To identify whether magnolol may induce cytotoxicity of GBM cells, we performed MTT assay on GBM8401 and BP-5 cells. As indicated in Figure 1A,B, superior cytotoxicity was found in GBM8401 cells as compared to BP-5 cells. The IC_50_ values of magnolol at 48 h on GBM8401 and BP-5 cells were 25 μM and 150 μM, respectively. Then, we further investigated whether magnolol may induce apoptosis of GBM8401 cells. As shown in Figure 1C, the annexin-V activation was increased after 48 h treatment with 50 μM magnolol. Furthermore, cleaved caspase-3 was also activated by magnolol treatment (Figure 1D). We further aimed to identify which apoptosis pathway is involved in magnolol-induced apoptosis; we tested both extrinsic and intrinsic apoptosis related markers after magnolol therapy by flow cytometry. In the cleaved caspase-8 activation results, magnolol may effectively induce the expression of cleaved caspase-8 as compared to the non-treatment group (Figure 1E). Both upstream of caspase-8, Fas, Fas-L, DR4 and DR5 were also found to be triggered by magnolol (Figure 1F–I). Magnolol markedly induced the activation of Fas in GBM8401 cells. In addition, we identified intrinsic related marker expression after magnolol treatment. As illustrated in Figure 1J, magnolol stimulated the elevation of caspase-9. The mitochondrial membrane potential loss (ΔΨm) was intensified after 48 h treatment with 50 μM magnolol (Figure 1K). The DR4 and DR5 protein expressions that validated by Western blotting was also increased after magnolol treatment (Figure 1L). These results indicated that magnolol not only induces markers involved in the extrinsic apoptosis pathway (i.e., Fas, Fas-L, DR-4, DR-5 and cleaved caspase-8) but also triggers the activation of intrinsic apoptosis-related factors (i.e., caspase-9 and ΔΨm loss). Taken together, magnolol may decrease the viability and induce the extrinsic/intrinsic apoptosis pathways of GBM cells.

### 3.2. Magnolol-Suppressed GBM Progression Is Associated with PKCδ/STAT3 Inactivation

**Figure 2:**
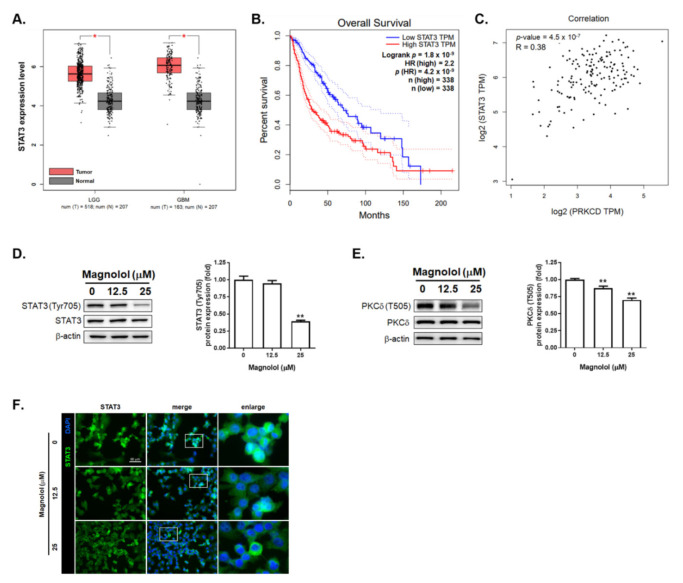
Inactivation effect of magnolol on the PKCδ/STAT3 signaling pathway of GBM cells. (**A**) STAT3 expression level in GBM and LGG tissues. (**B**) The overall survival of glioma patients between high and low STAT3 expression level. (**C**) The Pearson correlation coefficient results between STAT3 and PKCδ in GBM patient samples. (**D**,**E**) The protein expression pattern and quantification results of STAT3 and PKCδ after magnolol treatment for 48 h. (**F**) The IF staining results of STAT3 nuclear translocation. Green fluoresce represents STAT3. Nuclei are also stained by DAPI. Full Western blotting images have been provided in supplementary material (Material S1). (* *p* < 0.05, ** *p* < 0.01 vs. 0 μM magnolol).

In Figure 2A, the expression level of STAT3 was relatively high in low-grade gliomas (LGG) and GBMs as compared to that in the normal tissue. A poor survival pattern was also found in patients with higher STAT3 expression levels (Figure 2B). As illustrated in Figure 2C, PKCδ (PRKCD) expression was positively correlated with STAT3 expression level in GBM patients. These results indicated that PKCδ/STAT3 signaling pathway may be an important target for GBM treatment. Thus, we further identified whether magnolol suppresses the activation of PKCδ/STAT3 signaling pathway in GBM. Our Western data indicated that the phosphorylation levels of STAT3 and PKCδ were reduced by treatment with magnolol (Figure 2D,E). Furthermore, the nuclear translocation of STAT3 in GBM8401 cells was effectively inhibited by magnolol (Figure 2F). To conclude, magnolol-suppressed GBM progression is associated with the suppression of PKCδ/STAT3 signaling pathway.

### 3.3. Magnolol-Diminished GBM Invasion and Migration Is Associated with PKCδ/STAT3 Suppression

**Figure 3:**
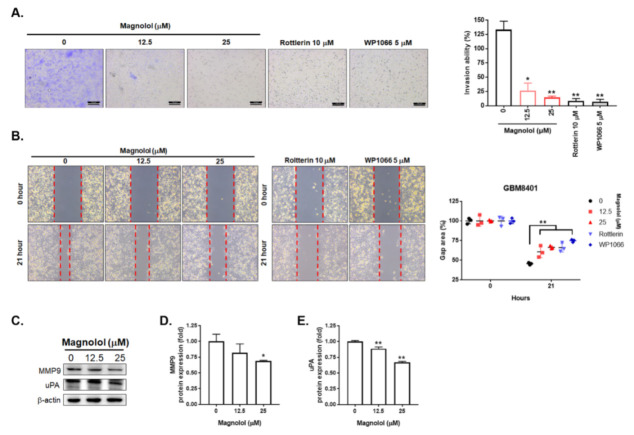
Suppression effect of magnolol on the invasion and migration ability of GBM cells. (**A**) The transwell invasion pattern and quantification data after 0, 12.5, 25 μM magnolol, 10 μM Rottlerin (PKCδ inhibitor) and 5 μM WP1066 (STAT3 inhibitor) treatment for 48 h in GBM8401 cells. (**B**) The wound healing pattern and quantification data in GBM8401 cells after various treatments as presented. (**C**–**E**) The Western blotting pattern and quantification results of MMP and uPA after magnolol treatment in GBM8401 cells. Full Western blotting images have been provided in supplementary material (Material S1). (* *p* < 0.05, ** *p* < 0.01 vs. 0 μM magnolol).

To further identify the effect of magnolol on GBM invasion and migration, we performed transwell invasion and wound healing assays. In Figure 3A, tumor invasion ability was markedly suppressed by magnolol, STAT3 inhibitor (WP1066) and PKCδ inhibitor (Rottlerin) after 48 h treatment. The invasion ability was also decreased in the treatment groups by 80–90%. Furthermore, magnolol, STAT3 inhibitor (WP1066) and PKCδ inhibitor (Rottlerin) treatment groups resulted in larger gap areas as compared to untreated group in the wound healing assay (Figure 3B). These results suggest that both invasion and migration activity of GBM cells was suppressed by magnolol. Finally, we also validated whether cell invasion associated genes in GBM are downregulated by magnolol. In Figure 3D–E, the protein expression levels of MMP-9 and uPA were decreased by treatment with magnolol. All above results indicated that the invasion and migration ability of GBM can be suppressed by magnolol.

## 4. Discussion

Death-receptor–ligand interaction and intracellular stress can initiate tumor apoptosis through the extrinsic and intrinsic pathways. In the process of death-receptor-mediated apoptosis, caspase-8 activation is required via cleavage; higher levels of cleaved caspase-8 have been associated with better clinical outcomes of GBM patients, rendering cleaved caspase-8 indicative of GBM prognoses [11,21]. Additionally, constitutive activation of the Fas/FasL signaling pathway may induce GBM cell apoptosis [22,23]. Our results showed that expression of cleaved caspase-8, Fas and Fas-L was significantly increased upon the treatment with magnolol (Figure 1E–G).

The dependence of magnolol-triggered GBM cell apoptosis on the intrinsic pathway in has been indicated by Cheng et al. [24]. Loss of mitochondrial membrane potential (ΔΨm), cytochrome C released from mitochondria and caspase-9 activated by apoptosome are biochemical hallmarks of the intrinsic apoptosis pathway [21,25]. In this study, we also found that loss of ΔΨm and expression of cleaved caspase-9 were both significantly induced by treatment with magnolol, suggesting that magnolol can induce GBM cell apoptosis not only through the extrinsic but also the intrinsic pathway (Figure 1J,K).

In this regard, STAT3 tyrosine 705 phosphorylation (STAT3 (Tyr705)) mediates the invasion of GBM cells by upregulating uPA, MMP-2 and MMP-9 [26,27], the decreased levels of STAT3 (Tyr705), uPA and MMP-9 by magnolol strongly indicate a counteraction from magnolol to GBM cell invasion. In correspondence to other previous findings that the decreased expression of STAT3 (Tyr705) was correlated with favorable survival in patients with GBM [28] while STAT3 silencing by siRNA reduced the invasion of GBM cells [29], the invasion of GBM8401 cells in this study was shown to be significantly suppressed by treatment with either magnolol or STAT3 inhibitor (Figure 3). According to the results of this study, that inhibition of STAT3 (Tyr705) was implicated to take part in magnolol-diminished invasion ability of GBM cells in reference to that magnolol-elicited inhibitory effect on GBM cell migration via N-cadherin downregulation [19].

Cell and animal models have been utilized to investigate the anticancer properties of magnolol [30]. Magnolol mediates cancer regression through the induction of apoptosis and the suppression of oncogenic pathways, such as transforming growth factor beta (TGF-β)/Samd, insulin-like growth factor 1 receptor (IGF-1R) and nuclear factor-kappaB (NF-κB), in hepatic, pancreatic and prostate cancers as well as cholangiocarcinoma [31,32,33,34]. In addition, magnolol elicits suppressive effects on the proliferation and migration of GBM cells by upregulating p21 and reducing N-cadherin expression [18,19]. In this study, we demonstrated that magnolol induced extrinsic (Figure 1E–I) and intrinsic (Figure 1J,K) apoptosis and abolished STAT3-mediated invasion in GBM cells (Figure 2 and Figure 3).

Protein kinase C isoenzymes (PKCs) are a family of serine/threonine kinases to mediate phosphorylation of downstream proteins that play important roles in several oncogenic signaling pathways [35,36]. PKCδ, a member of PKCs, has been reported to potentiate STAT3 activation and result in GBM cell invasiveness [37]. The invasion of GBM8401 cells, however, was significantly attenuated by PKCδ inhibitor (Figure 3). In addition to the positive correlation between the PKCδ expression and STAT3 expression in patients with GBM (Figure 2C), the alleviation of PKCδ phosphorylation by magnolol implies that suppression of PKCδ phosphorylation may be linked to magnolol-inhibited STAT3 activation in GBM (Figure 3).

## 5. Conclusions

Taken together, this study demonstrated that magnolol triggers apoptosis pathways and suppresses PKCδ/STAT3 activation in GBM cells. The induction of apoptosis through the extrinsic and intrinsic pathways as well as PKCδ/STAT3 inactivation, respectively, correlated with magnolol-elicited growth and invasion inhibition of GBM cells.

## Data Availability

The original contributions presented in the study are included in the article/Supplementary Material. Further inquiries can be directed to the corresponding author.

## Supplementary Materials

The following are available online at www.mdpi.com/article/10.3390/life11121399/s1, Material S1: Full Western Blot images.
