# Supplementary material for: Magnolol Induces the Extrinsic/Intrinsic Apoptosis Pathways and Inhibits STAT3 Signaling-Mediated Invasion of Glioblastoma Cells
Source: Life (Basel). 2021 Dec 14;11(12):1399. doi: 10.3390/life11121399 (PMC8706091; doi:10.3390/life11121399)
Supplement: Supplementary file 1 [file life-11-01399-s001.zip › life-1470377-supplementary.pdf]

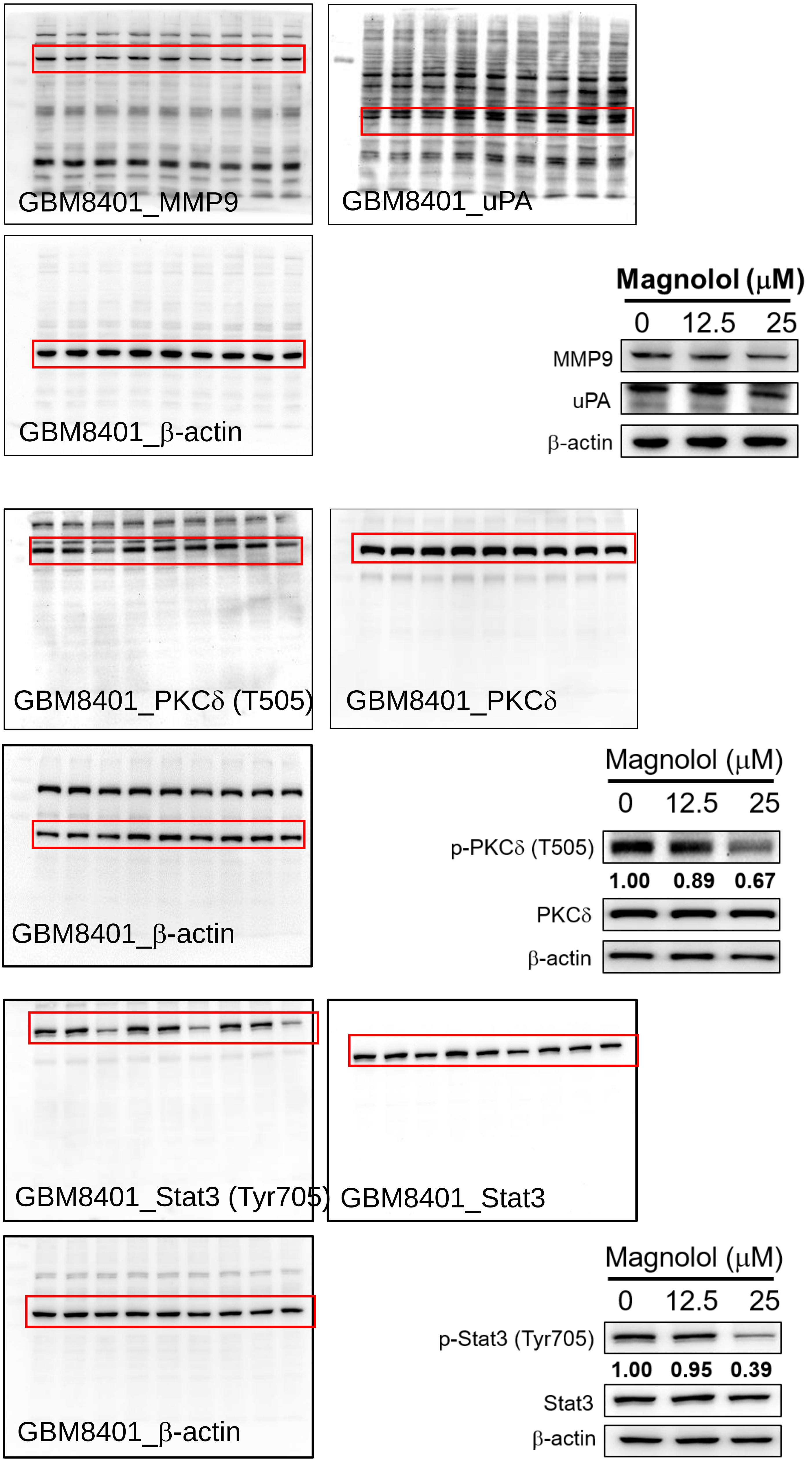
GBM8401_MMP9
GBM8401_uPA
GBM8401_β-actin
Magnolol (μM)
0 12.5 25
MMP9
uPA
β-actin
GBM8401_PKCδ (T505)
GBM8401_PKCδ
GBM8401_β-actin
Magnolol (μM)
0 12.5 25
p-PKCδ (T505)
1.00 0.89 0.67
PKCδ
β-actin
GBM8401_Stat3 (Tyr705)
GBM8401_Stat3
GBM8401_β-actin
Magnolol (μM)
0 12.5 25
p-Stat3 (Tyr705)
1.00 0.95 0.39
Stat3
β-actin

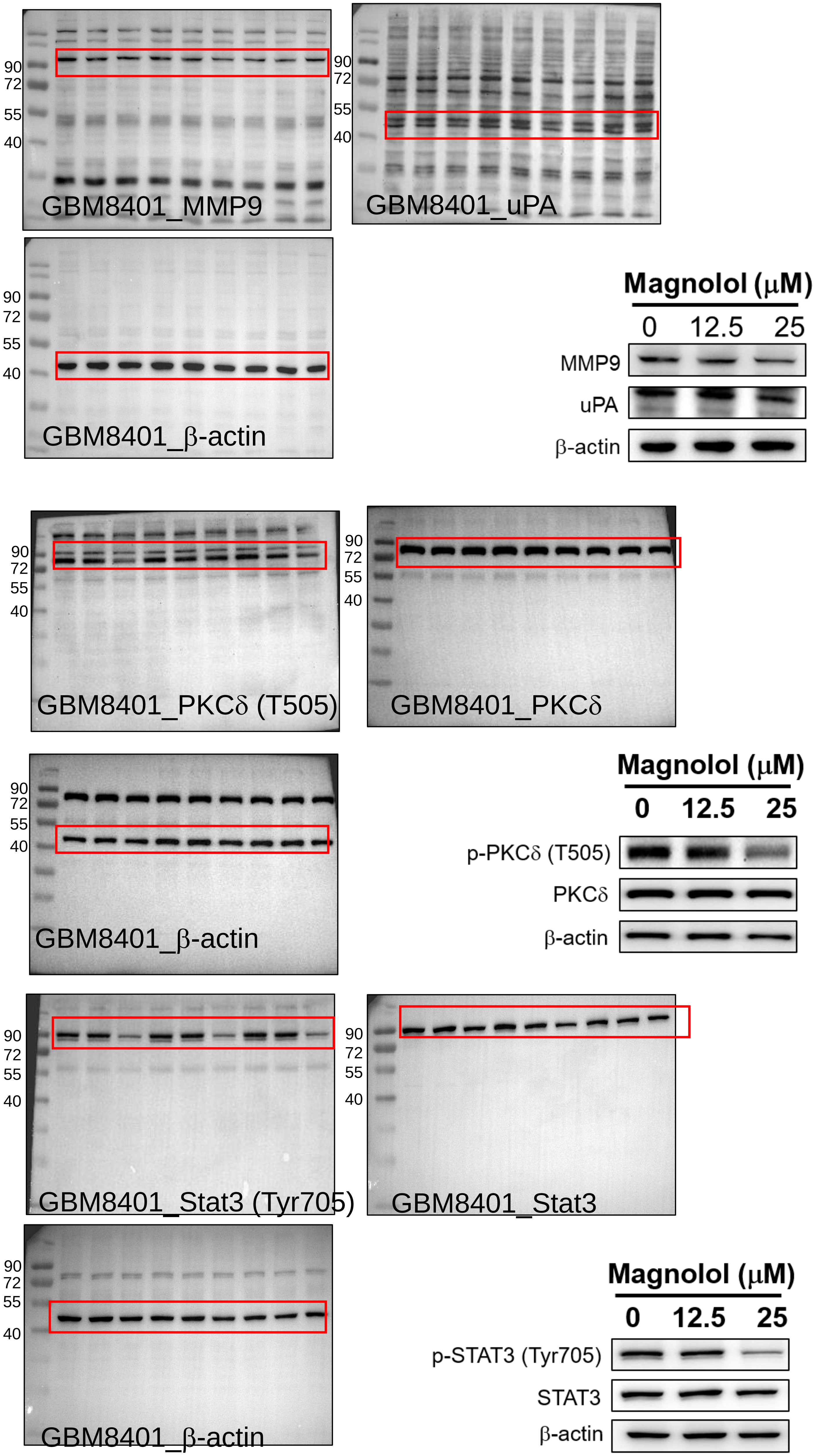

90
72
55
40
GBM8401_MMP9
90
72
55
40
GBM8401_uPA
90
72
55
40
GBM8401_β-actin
Magnolol (μM)
0
12.5
25
MMP9
uPA
β-actin
90
72
55
40
GBM8401_PKCδ (T505)
90
72
55
40
GBM8401_PKCδ
90
72
55
40
GBM8401_β-actin
Magnolol (μM)
0
12.5
25
p-PKCδ (T505)
PKCδ
β-actin
90
72
55
40
GBM8401_Stat3 (Tyr705)
90
72
55
40
GBM8401_Stat3
90
72
55
40
GBM8401_β-actin
Magnolol (μM)
0
12.5
25
p-STAT3 (Tyr705)
STAT3
β-actin

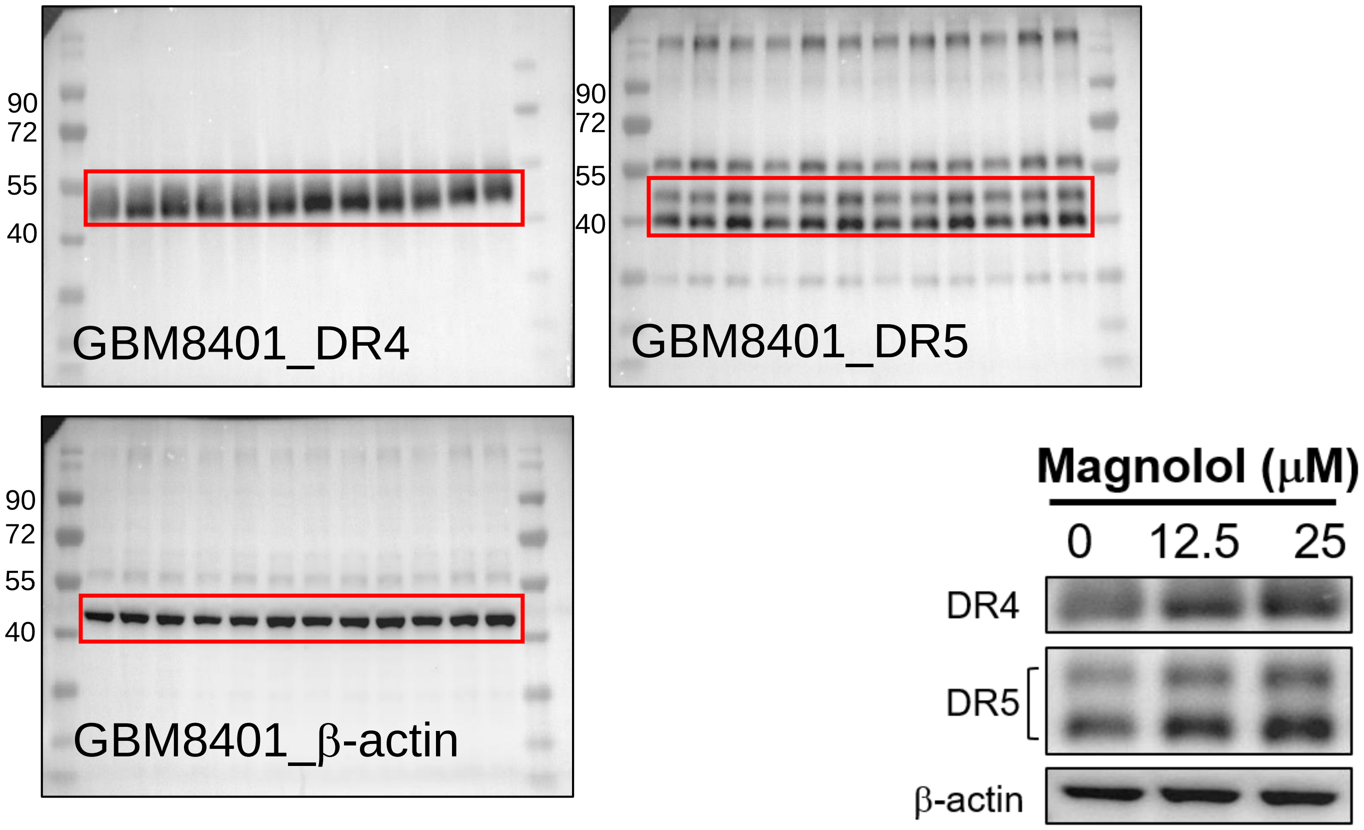

p-PKC(T505)	Magnolol 0uM	Magnolol 12.5uM	Magnolol 25uM
N1	1.182859	1.104385	0.734945
N2	0.903166	0.813739	0.698253
N3	0.913975	0.762721	0.585456
p-STAT3(Tyr705)	Magnolol 0uM	Magnolol 12.5uM	Magnolol 25uM
N1	0.894428	0.88964	0.425852
N2	1.065182	0.921425	0.385406
N3	1.04039	1.029325	0.366758
MMP9	Magnolol 0uM	Magnolol 12.5uM	Magnolol 25uM
N1	1.080446	0.718993	0.697082
N2	0.919554	0.918361	0.684845
uPA	Magnolol 0uM	Magnolol 12.5uM	Magnolol 25uM
N1	1.011084	0.871061	0.654434
N2	0.988916	0.905649	0.679247

DR4	Magnolol 0uM	Magnolol 12.5uM	Magnolol 25uM
N1	1.016684591	1.166600993	1.395462534
N2	0.864668176	1.136984946	1.17744816
N3	1.028767651	1.155784592	1.265775472
N4	1.089879582	1.20300559	1.217432504
DR5	Magnolol 0uM	Magnolol 12.5uM	Magnolol 25uM
N1	1.002716368	1.234650576	1.500662785
N2	0.993136999	1.122444222	1.363708379
N3	1.004146632	1.08467696	1.39184389
N4	0.983370177	1.228535669	1.251401131
N5	1.016629823	1.160115212	1.235530052
